# Genetic analysis of disqualifying traits in Nellore cattle

**DOI:** 10.1007/s11250-026-04971-5

**Published:** 2026-03-10

**Authors:** Fernanda Ripel Salgado, Amauri Felipe Evangelista, Juliana Cristina Sesana, Rodrigo de Almeida Teixeira, Laila Talarico Dias

**Affiliations:** 1 Graduate Program in Animal Science, Federal University of Paraná (UFPR), Curitiba, PR 80035‑010 Brazil; 2 State University of Goiás (UEG), Campos Belos, GO 73840-000 Brazil; 3 Program for Evaluation and Identification of Young Sires (PAINT®), Sertãozinho, Brazil; 4 Department of Animal Science, and Graduate Program in Animal Science, UFPR, Curitiba, PR Brazil

**Keywords:** Beef cattle, Functional trait, Genetic correlation, Bayesian inference, Paternal lineage

## Abstract

Morphological defects in beef cattle can compromise animal welfare, functionality and reproductive efficiency, generating economic losses and reducing longevity. Understanding their genetic basis is essential to support selection decisions and prevent the dissemination of defects in breeding programs. This study aimed to estimate the genetic parameters for defects of feet and legs (FL), mouth (MO), chamfer (CH), racial characterization (RC), depigmentation (DP), umbilical hernia (UH), testicular hypoplasia (TH) and navel (NV), evaluated as binary traits at 18 months of age, in Nellore cattle. The data was provided by the CRV Lagoa - PAINT^®^ beef cattle genetic breeding program, which belonged to the company until 2020. The components of (co)variance and breeding values were estimated by Bayesian inference using Animal Models. The genetic correlation between FL and yearling weight (YW) was obtained through a two-trait linear-threshold model. Posterior heritabilities for FL, MO, CH, RC, DP, UH, TH, NV and YW were 0.16 ± 0.03; 0.23 ± 0.04; 0.19 ± 0.03; 0.39 ± 0.04; 0.69 ± 0.04; 0.38 ± 0.12; 0.19 ± 0.04; 0.53 ± 0.04 and 0.45 ± 0.01, respectively. When lineage effect was considered fixed, all traits showed high heritability (> 0.40). The genetic correlation between FL and YW was negative and of low magnitude (-0.06 ± 0.05 without lineage; -0.01 ± 0.09 with lineage). The estimated genetic trends for FL, MO, CH, RC, DP, TH and NV declined over the years, indicating a gradual reduction of their incidence due to selection, while UH presented unstable pattern. Overall, the results indicated that including disqualifying traits in breeding programs can lead to reduced occurrence of morphological defects in Nellore herds.

## Introduction

Beef cattle farming is an activity carried out in all regions of Brazil and is a major component of the national economy. Over time, several investments have been made by producers, companies, and research institutions to increase the efficiency and profitability of national meat production (Neves et al. 2019). Traditionally, beef cattle breeding programs in Brazil use weights measured at different ages as selection criteria (Silveira et al. 2021).

In addition, the genetic evaluation of beef cattle in Brazil focuses on the selection of a series of economically important traits, such as production, precocity, fertility, carcass, and feed efficiency through the use of selection indexes (Bessa et al. 2021; Portes et al. 2021; Lôbo et al. 2023). In 1995, the Brazilian Ministry of Agriculture and Livestock (MAPA) started the CEIP (Special Certificate of Identification and Production), in which the 20% of genetically superior individuals, evaluated in their respective breeding programs, for economically important traits are able to receive this recognition. However, individuals eligible to receive CEIP later undergo two other evaluations so that they can actually be used as breeders: andrological testing and evaluation of traits of morphological function.

According to Silva et al. (2023), the CEIP guarantees a differentiated market value for animals and their products, and even animals of high genetic value and candidates for CEIP, can be disqualified and slaughtered if they present any morphological alteration. Among the most frequently observed morphological defects are problems with feet and legs, pigmentation, racial characterization, chamfer, mouth, umbilical hernia, testicular hypoplasia and navel. These defects can affect the performance of the animals, favoring the appearance of pain, diseases and, in more serious cases, lead to death. Therefore, the use of these animals in reproduction may harm production, because if these traits present moderate to high heritability, they will be transmitted to future generations (Silva et al. 2022; Souza et al. 2024).

For this reason, early identification and selection of animals that do not have defects is relevant for beef cattle. A more efficient approach to reduce the occurrence of these defects in the beef cattle population would be to perform genetic evaluations of these traits and use the expected breeding values as selection criteria, highlighting the importance of studying the magnitude of genetic variability and heritability of the trait, as well as possible genetic associations with other traits in selection (Vargas et al. 2017).

However, records of morphological defects are not common and when there are, it is not possible to correctly identify the severity of the animal’s impairment, since the information contained in the form only reports that the animal presented a certain defect. This can justify the difficulty in finding articles in the literature about the estimation of genetic parameters for morphological defects and the genetic correlation between certain defects and productive and reproductive traits in beef cattle. Therefore, according to Marcondes et al. (2005), it is important to estimate genetic parameters for disqualification defects in cattle to later use them as selection criteria. In addition, identifying sire lines that have defects is important, since the lines have a strong influence on the decision to choose which sire will be used in the breeding season.

Therefore the objectives of this study were to estimate (1) the genetic parameters for disqualifying morphological traits, considering or not the effect of paternal lineage, (2) genetic correlation between feet and legs against yearling weight and (3) the genetic trends for the traits studied.

## Materials and methods

Approval from the Animal Care and Use Committee of the Federal University of Paraná (UFPR) was not required for this study, as the data were obtained from an existing database of Nellore cattle.

### Data

Yearling data from 411,380 males and females, born between 1994 and 2017, belonging to the database of the PAINT^®^ (Program for Evaluation and Identification of Young Sires) beef cattle breeding program, whose 207 participating farms are located in the Northeast, North, Southeast and Midwest regions of Brazil, were used.

The animals were weighed at yearling between 340 and 670 days of age (age range required by the program for yearling assessment), grouped according to month of birth and sex, and evaluated by the program technicians using visual method in a binary way for the presence (1) or not (0) of problems in feet and legs (FL), mouth (MO), chamfer (CH), racial characterization (RC), depigmentation (DP), umbilical hernia (UH), testicular hypoplasia (TH – evaluated only in males) and navel (NV).

For all traits (FL, MO, CH, RC, DP, UH, TH and NV) the contemporary groups (CG) were formed by the variables: year, farm and season of birth, sex (except for TH), weaning farm, yearling farm, weaning management group and yearling management group, and CG with less than 10 animals were excluded, as well as those groups without phenotypic variability (in which all animals had the same score attribution value). The relationship matrix used contained data from 1,132.623 animals, which included 411,380 cows with phenotypes, 11,286 sires, and 526,091 dams. Data editing was performed using SAS 9.4 software (SAS Institute, 2014).

### Estimates of genetic parameters

After consistency analysis and data editing, the variance components and estimated breeding values (EBV) for each animal were obtained through univariate analyses, under a Bayesian approach. The analyses were performed through the iterative process of Gibbs sampling using the GIBBSF90 + software (Misztal et al. 2022), fitting an animal model of threshold. To evaluate a possible response to the correlated selection of yearling weight in feet and legs (FL), a two-trait analysis was performed under a linear-threshold animal model (Vargas et al. 2017).

The statistical model used in the first analysis can be described in matrix notation as:

*Y=Xβ+Za+e,*

where ***Y*** is the vector of observations for the traits, **β** is the vector of fixed effects (including the classificatory effect of the contemporary group, region, and the weight at yearling as linear and quadratic covariates), **a** is the vector of direct additive genetic effects, and **e** is the vector of random residual effects, where **X** and **Z** are the incidence matrices relating the elements of ***Y*** to the vectors **β** and **a**, respectively. The following assumptions were assumed: a^∼^N (0, A**σ²**_**a**_) and e^∼^N (0, **Iσ²**_**e**_), where **A** is the numerator relationship matrix, **σ²**_**a**_ is the additive genetic variance, **I** is an identity matrix and **σ²**_**e**_ is the residual variance. Since **σ²**_**e**_ is not estimable in threshold models for binary features (Gianola and Foulley 1983), the parameterization 2e = 1 was used (Sorensen and Gianola 2007).

The thresholds of the binary features under study can be defined as:

$${{\{ }}{\mathrm{y}_\mathrm{i}} = 0\,\mathrm{se}\,{\mathrm{l}_\mathrm{i}} \leq \mathrm{t}_1;{\mathrm{y}_\mathrm{i}} = 1\,\mathrm{se}\,\mathrm{l}_\mathrm{i} > \mathrm{t}_1{\mathrm{\} }}$$

where: y_i_ is the score of the i-th animal, t corresponds to the threshold that defines, on the underlying scale, the exclusive categories of traits (0 or 1) (Gianola and Foulley 1983).

Sample chains (Gibbs chains) with a length of 1,000,000 cycles were originated, with initial discard (burn-in) of 250,000 samples and a storage interval (thin) of 25 samples, except for wry nose and the correlation between yearling weight and FL, which used burn-in of 300,000 samples. Convergence was verified based on the criterion proposed by Geweke (1992) and Heidelberger and Welch (1983) through the BOA package (Smith 2007) available in the statistical software R (R Core Team 2022).

Genetic trends were estimated by linear regression of the subsequent means of the breeding values for each trait throughout the year of birth of animals with known paternity. The subsequent means of the estimated breeding values for each trait were standardized in terms of the subsequent mean of the genetic standard deviation.

Given the importance of choosing which sire to use in mating to avoid inbreeding, it is also relevant to pay attention to the possibility that these sires contribute to the occurrence of resulting progeny defects. Then, the pedigree column was created in the data file based on the pedigree file (animal, sire and dam) using the PROC SQL procedure of the SAS software (SAS Institute 2018).

For the formation of the lineages, maternal and paternal, grandfathers and great-grandfathers were initially identified. From this, the frequency of defects per great-grandparent was calculated and thus selected the animals with the highest number of offspring with defects. These animals were considered the ancestors of the lineage, and individuals that had sire A as father, grandfather or great-grandfather were included in lineage 1, animals that had sire B as father, grandfather or great-grandfather were included in lineage 2 and so on. The number of animals per line is shown in Table 1.

**Table 1:** Total number of descendants in each line and percentage of defects evaluated in Nellore cattle

Lineage	Traits							
	Nº	FL	MO	CH	RC	DP	TH	NV
1	3.439	1.57%	0.41%	1.86%	0.35%	0.41%	0.47%	0.41%
2	7.017	1.60%	0.23%	1.36%	0.71%	0.48%	1.09%	0.35%
3	4.578	1.70%	0.66%	1.42%	1.62%	2.49%	1.77%	3.06%
4	4.766	1.38%	1.07%	2.01%	1.51%	1.13%	1.15%	1.57%
5	4.381	2.72%	0.80%	0.94%	0.57%	1.44%	0.89%	0.21%
6	3.314	1.81%	0.78%	5.10%	1.03%	0.39%	1.21%	1.39%
7	4.581	1.81%	0.35%	1.40%	1.27%	0.52%	0.94%	0.63%
8	7.209	2.62%	0.17%	0.82%	0.58%	0.17%	1.12%	0.17%
9	6.468	2.80%	0.56%	1.75%	1.39%	0.57%	1.65%	0.63%
10	6.593	3.25%	0.46%	1.64%	0.42%	4.44%	1.05%	0.26%
11	8.842	2.86%	0.69%	3.03%	1.21%	2.11%	1.27%	0.81%
12	10.623	2.33%	0.29%	1.92%	0.83%	1.69%	1.00%	0.72%
13	4.411	1.44%	0.81%	1.51%	0.90%	0.29%	1.40%	1.67%

Nº: Number of animals: feet and legs (FL), mouth (MO), chamfer (CH), racial characterization (RC), depigmentation (DP), testicular hypoplasia (TH), and navel (NV)

After the formation of the lines, the genetic analysis was carried out according to the methodology and model described above. In this analysis, the lineage effect was included as fixed. Thus, the fixed effects considered were: the contemporary group (CG), region and lineage. It is worth noting that, only for the feet and legs trait, the linear and quadratic effects of yearling weight were considered in the analysis model as a covariate.

## Results

Table 2 presents the descriptive structure of the data used in the quantitative analysis of the disqualification traits obtained after editing the data set. The number of observations differed considerably among traits, ranging from 8,054 records for umbilical hernia (UH) to 143,714 records for chamfer (CH). The occurrence of animals that presented defects was 4.58%, 2.35%, 3.81%, 4.62%, 3.26%, 2.06%, 4.37% and 3.88%, respectively for feet and legs (FL), mouth (MO), chamfer (CH), racial characterization (RC), depigmentation (DP), umbilical hernia (UH), testicular hypoplasia (TH) and navel (NV).

**Table 2:** Descriptive structure of the data of disqualification traits in Nellore cattle

Traits	Nº	GC	0	1	Occurrence
Feet and Legs	136.842	2.959	130.579	6.263	4.58%
Mouth	65.359	1.150	63.825	1.534	2.35%
Chamfer	143.714	3.060	138.233	5.481	3.81%
Racial Characterization	103.422	2.183	98.641	4.781	4.62%
Depigmentation	116.866	2.342	113.057	3.809	3.26%
Umbilical Hernia	8.054	1.48	7.888	166	2.06%
Testicular Hypoplasia	87.017	1.954	83.212	3.805	4.37%
Navel	95.038	1.942	91.355	3.683	3.88%

Nº= Number of animals; CG= Contemporary group; 0 = no defects; 1 = defects

**Fig. 1:**
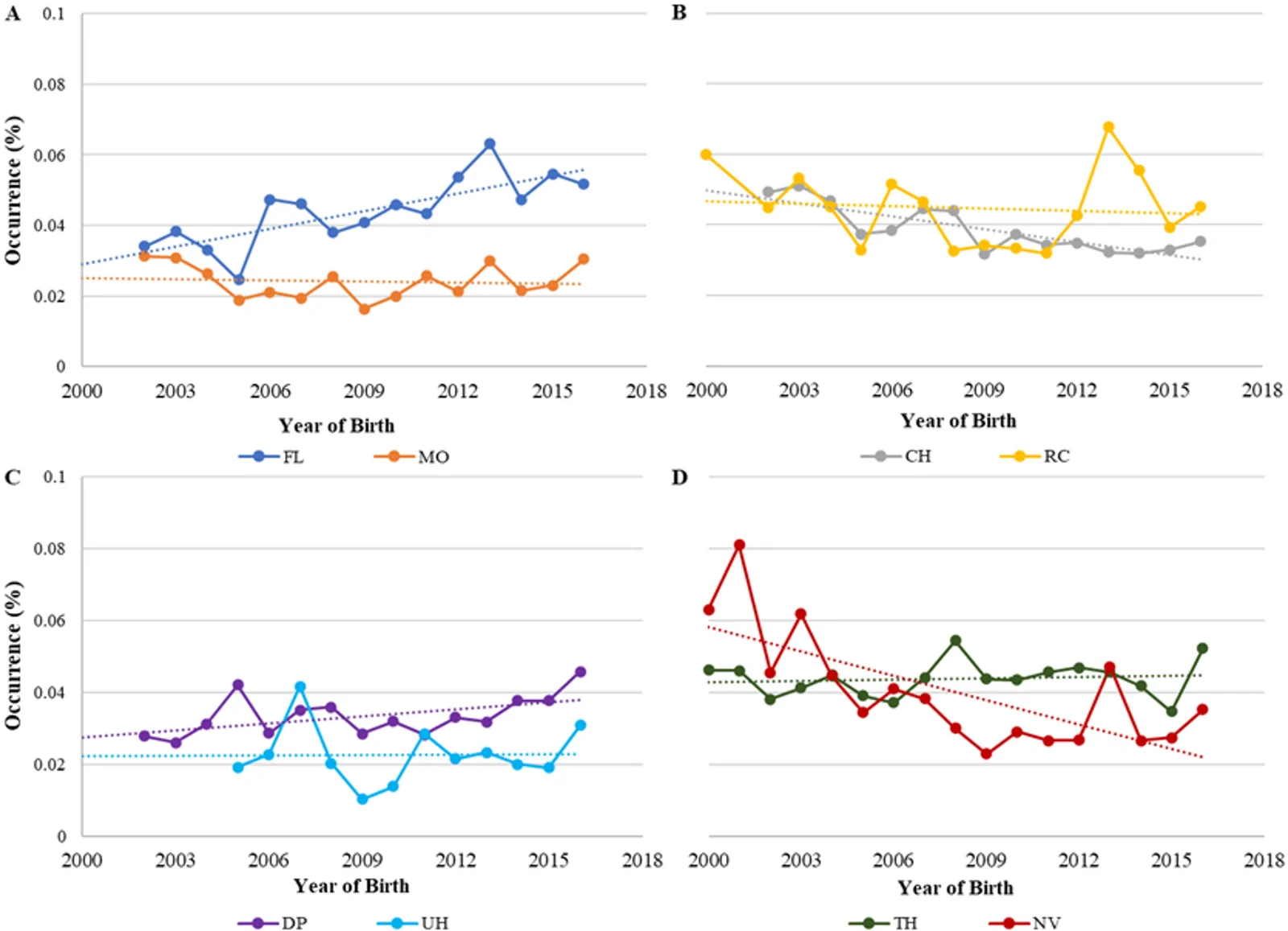
Occurrence of defects in Nellore cattle evaluated at yearling by year of birth. Subfigure letters indicate the traits shown: (**A**) feet and legs (FL) and mouth (MO), (**B**) chamfer (CH) and racial characterization (RC), (**C**) depigmentation (DP) and umbilical hernia (UH), and (**D**) testicular hypoplasia (TH) and navel (NV)

The mean frequency of the disqualification traits showed different behavior over the years in the population studied, reaching maximum values of 14.95% for racial recharacterization in 2000, and minimum values for Umbilical Hernia (1.09%) in 2009 (Fig. 1). It is noteworthy that over the years the average frequency of FL, DP and TH have increased over the years, while CH and NV have shown a declining trend. The other disqualifying traits (MO, RC and UH) showed stable behavior.

Table 3 presents the a posteriori estimates of the heritability coefficients for the disqualifying traits and yearling weight (YW) in Nellore cattle. In general, the lineage affected the genetic parameters included in the animal model.

**Table 3:** Estimates of additive genetic variance (σ²a), residual (σ²r) and heritability (h^2^) for disqualifying traits and weight in Nellore cattle at yearling, considering or not the effect of paternal lineage in the analysis model

Traits	No lineage			
	σ²a	σ²*r*	h²±PSD	CI95%
Feet and Legs	0.190	1.007	0.16 ± 0.03	0.10–0.21
Mouth	0.299	1.004	0.23 ± 0.04	0.13–0.31
Chamfer	0.243	1.006	0.19 ± 0.03	0.14–0.25
Racial Characterization	0.662	1.005	0.39 ± 0.04	0.30–0.48
Depigmentation	2.301	1.003	0.69 ± 0.04	0.61–0.77
Umbilical Hernia	0.685	1.004	0.38 ± 0.12	0.17–0.61
Testicular Hypoplasia	0.237	1.007	0.19 ± 0.04	0.12–0.27
Navel	1.171	1.004	0.53 ± 0.04	0.45–0.62
Yearling Weight	317.17	383.64	0.45 ± 0.01	0.44–0.46
	**With Lineage**			
Feet and Legs	0.858	1.010	0.44 ± 0.10	0.24–0.61
Mouth	4.102	0.994	0.74 ± 0.11	0.51–0.93
Chamfer	0.819	1.010	0.43 ± 0.10	0.23–0.65
Racial Characterization	1.854	1.007	0.58 ± 0.16	0.27–0.85
Depigmentation	7.118	0.979	0.85 ± 0.06	0.74–0.93
Umbilical Hernia	-	-	-	-
Testicular Hypoplasia	1.070	1.008	0.48 ± 0.13	0.23–0.72
Navel	3.980	0.997	0.72 ± 0.13	0.47–0.94
Yearling Weight	397.90	355.38	0.53 ± 0.02	0.49–0.57

PSD: Posterior standard deviation; CI: Credibility interval at 95%

For the analyses in which the lineage effect was not included, the heritability estimates for the disqualifying traits ranged from low to high magnitude, with values of 0.16 ± 0.03 for feet and legs, 0.23 ± 0.04 for mouth, 0.19 ± 0.03 for chamfer, 0.39 ± 0.04 for racial characterization, 0,69 ± 0,04 for depigmentation, 0.38 ± 0.12 for umbilical hernia, 0.19 ± 0.04 for testicular hypoplasia, and 0,53 ± 0,04 for navel defects. The heritability for yearling weight was 0.45 ± 0.01.

When the lineage effect was considered as fixed in the model, the heritability estimates were 0.44 ± 0.10 for feet and legs, 0.74 ± 0.11for mouth, 0.43 ± 0.10 for chamfer, 0,58 ± 0,16 for racial characterization, 0.85 ± 0.06 for depigmentation, 0.48 ± 0.13 for testicular hypoplasia, and 0.72 ± 0.13 for navel (Table 3). The heritability for yearling weight was 0.53 ± 0.02. It was not possible to estimate the heritability coefficient for umbilical hernia, because the amount of information for this trait in this data file was very small.

In addition to the heritability coefficients, the genetic correlation between feet and legs (FL) and yearling weight (YW) was also estimated. For the analysis without the lineage effect, the genetic correlation was low and negative (–0.06 ± 0.05), while for the analysis including lineage as a fixed effect, the estimate was − 0.01 ± 0.09.

**Fig. 2:**
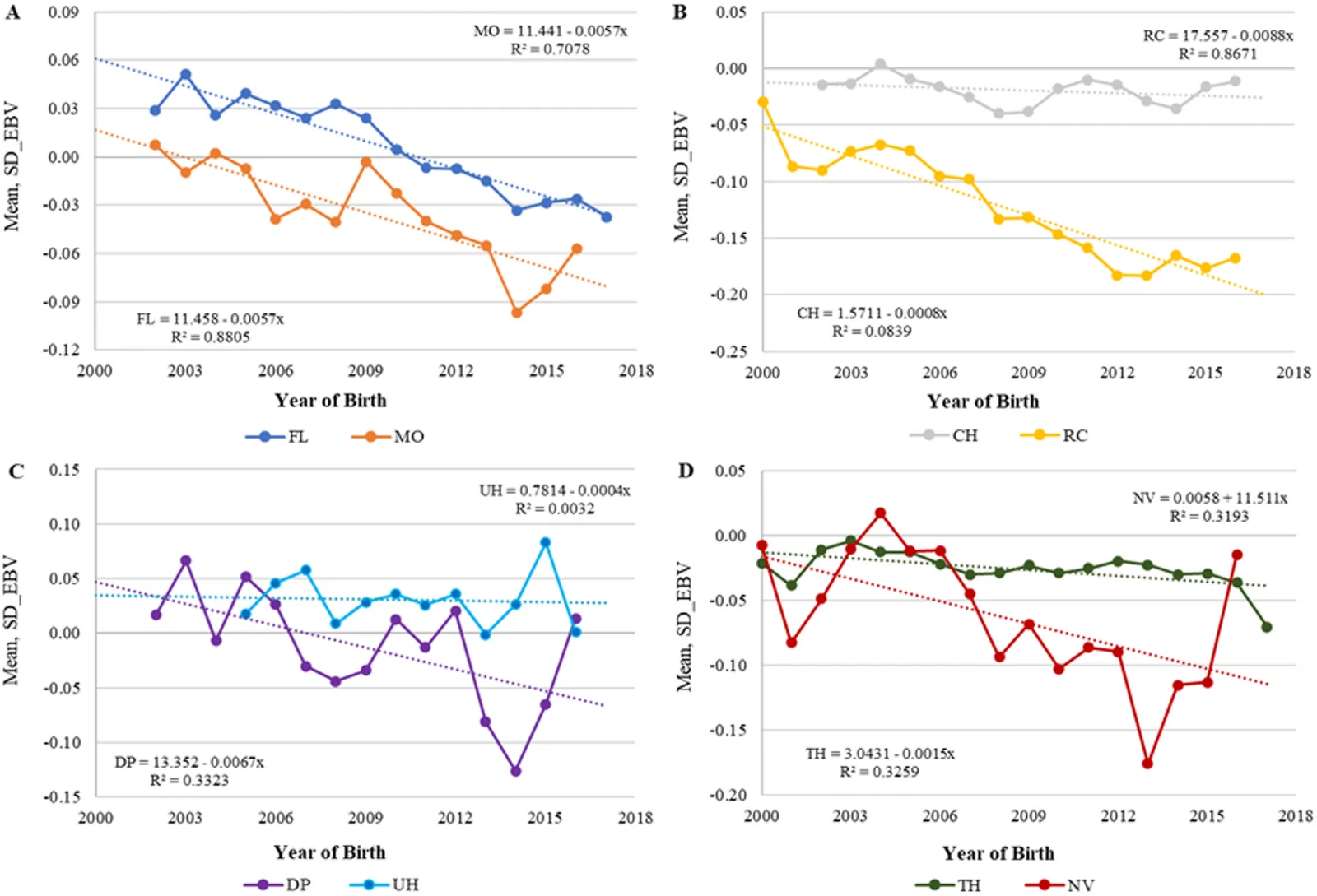
Genetic trends of the posterior means of the estimated breeding values (EBV) per year of birth for disqualifying traits evaluated at yearling in Nellore cattle. Subfigure letters indicate the traits shown: (**A**) feet and legs (FL) and mouth (MO), (**B**) chamfer (CH) and racial characterization (RC), (**C**) depigmentation (DP) and umbilical hernia (UH), and (**D**) testicular hypoplasia (TH) and navel (NV). Mean, SD_EBV = posterior mean of the EBV expressed as standard deviation (SD)

The genetic trends for morphological defects are shown in Fig. 2.

Annual genetic trends for feet and legs, mouth, chamfer and racial characterization were, respectively, -0.0057; -0,0057; -0.0008 and − 0.0088. The annual genetic trends for depigmentation, umbilical hernia, testicular hypoplasia and navel were, respectively, -0.0067; -0,0004; -0.0015 and − 0.0058. The coefficients of determination used to regress the mean breeding values for the disqualifying traits were all higher than 0.30 (except for the bevel and umbilical hernia traits).

## Discussion

The occurrence of animals that had defects was higher than 2%, which can result in economic losses to farmers, as animals with defects are usually unable to perform their productive and reproductive functions satisfactorily (Vargas et al. 2017). In addition, in Brazil, animals evaluated at yearling, which presented defects and those genetically inferior, cannot be candidates for the Special Certificate of Identification and Production (CEIP).

The animals that were classified among the 20% genetically superior and that would be able by genetic evaluation to receive the CEIP, still undergo evaluation of disqualification defects and andrological examination. If the animal has a defect and/or does not meet the requirements of the andrological examination, it will be disqualified. The issue is that the list of animals eligible for the CEIP is fixed and is not reviewed, which prevents other animals from receiving the certificate. Imagine that after the genetic evaluation 100 young sires are able to receive the CEIP, of which 20 did not pass the requirements of the andrological examination and/or presented some disqualifying defect, only 80 individuals will receive certification from the Ministry of Agriculture and Livestock (MAPA) and can be marketed as genetically superior sires. Even though the sire ranked 101st passed the andrological examination and did not present a disqualifying defect, it could not receive the CEIP due to legislation of MAPA.

Among the traits evaluated, the occurrence of animals with feet and legs problems (4.58%) and testicular hypoplasia (4.37%) was similar to the values reported of 4.50% and 4.61%, by Vargas et al. (2017) and Neves et al. (2019), respectively. The authors highlighted that the economic losses resulting from animals with disqualification defects are substantial, since young sires that do not receive CEIP cannot be sold as genetically superior sires.

Umbilical hernia was the least frequent defect (2.06%) in the population studied, this may be associated with the fact that this defect is observed early in the animal’s life, quickly leading to disposal. In addition, it is possible that this defect is more frequent in the evaluation performed at weaning, and in the present study the data analyzed refer to the yearling evaluation. Herrmann et al. (2001) identified the occurrence of congenital umbilical hernia of 1.8% in Fleckvieh calves in Germany, a value close to that found in the present study. The authors recommended using sires that were examined when they were still calves in breeding to confirm that the umbilical ring was indeed closed. In the literature it was not possible to find references about the occurrence of animals with mouth defects, chamfer, racial characterization, depigmentation and navel.

Figure 1 shows that the occurrence of FL and TH defects has increased over the years. Silva et al. (2023) reported that the increase in the occurrence of FL in recent years may have been influenced by the subjectivity of the assessment and should be observed with caution, as field technicians gain experience, they tend to be more demanding in the assessment. It is important to emphasize that even with the advancement in management practices to improve the performance of affected animals, the use of animals with reproduction defects may contribute to expanding the occurrence of these problems in herds (Neves et al. 2019), which is undesirable.

### Heritability

Identifying whether there is sufficient genetic variation for disqualifying traits to use them as a selection criterion in beef cattle herds is not common, given the difficulty in obtaining zootechnical files that have this information available. In addition, obtaining lineage information and using it in the analysis procedure is also uncommon, but in this database it was possible.

The heritability estimate for FL defects was of low magnitude (0.16 ± 0.03), indicating that a significant portion of the observed phenotypic variation can be attributed to environmental factors, and that the genetic gain obtained through direct selection is likely to be small and slow. A similar result was described by Vargas et al. (2018) in Nellore cattle, who reported a value of 0.16 ± 0.03, emphasized that this trait is polygenic with many genes explaining a relatively small proportion of the total genetic variance of traits, and that these findings suggest that FL should respond to selection.

However, previous studies with Nellore cattle (Lima et al. 1989; Koury Filho et al. 2003) reported higher heritability coefficients for FL, ranging from 0.37 to 0.45. However, it is essential to note that these authors evaluated FL through scores (evaluation scores ranging from 1 to 5), while in the present study, the trait was evaluated in a binary way, and factors such as sample size and methodology (including the use of restricted maximum likelihood) also contributed to the observed variations.

While for mouth problems, the heritability coefficient was 0.23 ± 0.04 and there are no studies in the literature for comparison, but such estimates indicate the possibility of genetic progress through selection. However, if there were a distinction between the observed defects, such as prognathism, it would be possible to better understand the genetic variability for this trait and obtain estimates that would more adequately indicate which traits respond to selection. Despite this, the non-use of sires that have mouth problems can help to reduce the appearance of this defect in herds.

The heritability estimate for chamfer defects was 0.19 ± 0.03, indicating greater environmental than genetic influence on this trait, so the response to selection will be slow. Lima et al. (1989) evaluated chamfer length in sires of the Nellore breed, as well as pole and ear, and estimated heritability close to zero. The authors concluded that the result obtained could be explained by the uniformity of the herd under study, which presented a breed pattern. In Brazil, although beef cattle producers are looking for more productive animals, racial standardization is still important.

The heritability estimate for racial characterization (RC) was 0.39 ± 0.04, indicating that it is possible to select animals for breed traits. In general, producers of track animals carry out selection for such traits, since they aim to meet all the requirements of the breed standard. Unlike our result, Boligon et al. (2016) estimated heritability of 0.14 ± 0.01 for RC and highlighted the possibility of limited genetic gain in this trait when used for selection for Nellore cattle. However, the authors reported that this trait was evaluated by means of a score, ranging from 1 (worst racial quality standard) to 5 (close to the racial quality standard), which may explain the difference between the estimated values, since in the present study RC was evaluated in a binary manner.

The high estimate of heritability for depigmentation (0.69 ± 0.04) suggests that this trait is influenced by genetic factors and, therefore, selecting animals that do not present depigmentation will be efficient to reduce the transmission of this defect. Therefore, it is possible to obtain genetic gains by selecting against depigmentation (Lima et al. 1989; Reimann et al. 2018), and that this trait can be included as a selection criterion to minimize this defect in the herd.

On the other hand, Souza et al. (2024), studying pigmentation in the eye area of Hereford and Braford cattle, estimated low magnitude heritability (0.195 ± 0.018) and concluded that moderate genetic gains for the trait are expected, even in the long term. In addition, the selection of animals with the best genetic potential for ocular pigmentation is a simple and viable alternative to reduce the occurrence of eye diseases. carcinoma in cattle, especially those intended for reproduction, as they remain in the herds for a longer period.

The moderate heritability coefficient for umbilical hernia (0.38 ± 0.12) estimated in this study indicates the possibility of responding to selection. A similar result (0.36) was reported by Herrmann et al. (2001) in Fleckvieh calves in Germany. The authors highlighted that there is probably more than one gene involved in the expression of this trait and that the occurrence could be reduced with early identification and selection.

Testicular hypoplasia (TH) is a morphological and functional reproductive disorder that affects sires around the world and, consequently, causes significant economic losses due to reduced fertility rates (Vargas et al. 2017). The condition is associated with decreased sperm concentration, a higher incidence of morphological abnormalities in spermatozoa, and, in severe cases, azoospermia (Silva et al. 2022). In the present study, the heritability estimate for testicular hypoplasia was 0.19 ± 0.04, similar to the value reported by Neves et al. (2019), who estimated heritability of 0.16 for this trait evaluated in a binary way in Nellore cattle. In the same study, the authors reported a negative correlation of -0.53 between testicular hypoplasia and scrotal circumference, indicating that animals with smaller scrotal circumference are more likely to have testicular hypoplasia, i.e., selection for scrotal circumference would reflect in the reduction of hypoplasia. Similarly, Silva et al. (2022) estimated heritability of 0.168 ± 0.023 for TH in Nellore cattle and emphasized that the variability is influenced by additive genetic effects, indicating that this occurrence can be reduced through selection in breeding programs.

The heritability estimate for the navel was of high magnitude (0.53 ± 0.04) indicating the possibility of genetic progress for this trait due to the high variation attributed to the additive genetic effect. This result is especially relevant for beef cattle raised on pastures, as correcting the size of the navel will improve the adaptation of these animals to the breeding environment. In addition, the selection and use of bulls with desirable genetic values for the navel should reduce economic losses resulting from injuries and traumas caused by vegetation, especially in animals with long and pendulous navels and foreskins, due to the inability to perform their reproductive functions satisfactorily (Souza et al. 2020).

In the literature, heritability estimates for the navel trait range from 0.06 to 0.70 (Lima et al. 1989, 2013; Viu et al. 2002; Campos et al. 2018; Silveira et al. 2021). The difference in the number of animals evaluated, breeds, evaluation methodology for obtaining the score, management and models used may justify the great variation in the magnitude of the estimates, but even the authors who estimated low values affirm that it is possible to obtain genetic gains.

The heritability estimate for yearling weight was of high magnitude (0.45 ± 0.01), showing that a significant portion of the observed phenotypic variation is attributable to the genetic factor. The results obtained agree with other studies with yearling Nellore cattle (Ribeiro et al. 2017; Portes et al. 2020; Watanabe et al. 2021; Barro et al. 2023), which corroborates the direct selection carried out for this trait by the most different beef cattle breeding programs (Soares et al. 2024).

The use of the lineage as a fixed effect resulted in higher values of direct heritability, suggesting that the inclusion of this effect helped in the identification of the genetic variability of the traits studied. In general, the estimates for the disqualifying traits were high and ranged from 0.44 ± 0.10 (FL) to 0.85 ± 0.06 (DP). These results suggest that it is possible to identify genetically superior sires and that some disqualifying defects are transmissible from one generation to another. From the identification of carrier breeders, it will be possible to avoid mating between animals that may transmit undesirable traits to the progeny.

Marcondes et al. (2007) analyzed the effect of lineage on the probability of cow permanence in Nellore herds and concluded that the family had an influence on the trait and that there was a contribution from the genes of the ancestors of the breed considered. In addition, the authors concluded that the identification of lineages and the differences between them is important to direct mating, avoid inbreeding, and promote greater genetic progress in traits.

So, directed mating programs would be a solution to reduce the occurrence of these defects in herds. These programs use the information of the animal itself and the pedigree to indicate the matings that would promote greater genetic gains, among the animals with higher genetic values to achieve the goal. By including information on defects, it is avoided to carry out matings between animals that have defects or that the lineage presents a greater probability of transmitting disqualifying traits. In addition, by considering the lineage, it will be possible to identify if there is an influence on defects and use this information to direct matings and reduce the occurrence of these problems.

### Genetic correlation

Considering the importance of the estimated genetic variation for defects in beef cattle, knowing its relationship with traits of economic interest is relevant. In this study, the genetic correlation between feet and legs as well as yearling weight was low and negative, with values of -0.06 ± 0.05 (file without lineage) and − 0.01 ± 0.09 (file with lineage), which indicates that there is no relationship between them. This correlation was higher than the value estimated in the literature of − 0.2311 ± 0.0076 by Silva et al. (2023) in Nellore cattle, emphasizing that animals with mobility difficulties due to congenital deformities in the feet and legs tended to gain less weight.

### Genetic tendency

The knowledge of the genetic trend of the disqualifying traits facilitates the identification of genetic progress over the period studied. Thus, it can be seen that the behavior of the genetic trend for the disqualification defects (Fig. 2) occurred in the desired direction, indicating that the animal selection strategies adopted by the animal breeding programs are promoting favorable changes, contributing to the genetic progress in these population traits, that is, they are decreasing over time. Similar results were reported by Vargas et al. (2017) and Silva et al. (2022) in a herd of Nellore cattle.

Vargas et al. (2017) highlighted that the genetic tendency for feet and legs occurred in the desired direction, and that this result may be due to indirect response to selection, since yearling weight was favorably correlated with feet and legs (score 1 to 5 - ranging from least ideal to most ideal). Silva et al. (2022) reported that the genetic tendency for testicular hypoplasia (TH) was in the desired direction, evidencing that there is continuous genetic progress for the trait, suggesting that the selection strategy of animals affected by TH promotes favorable genetic alterations. It is important to highlight that no genetic tendency for defects of the mouth, chamfer, racial characterization, depigmentation, umbilical hernia and navel was found in the literature to be used as a comparison.

Therefore, this information obtained in the present study may help mating programs aimed at identifying the best matings among animals, combining genetic values and pedigree, to maximize the genetic gain of the herd and, by adding information on defects, minimize their occurrence in national herds. Thus, a better understanding of defects in beef cattle can reduce the occurrence of this problem in Nellore herds and increase production and reproductive efficiency.

In conclusion, the study found that considering the disqualifying traits in directed mating programs may reduce the occurrence of morphological defects in Nellore herds. In addition, although there is some genetic influence on feet and legs yearling weight, the genetic association between these traits is not predominantly explained by genetic factors. The selection strategies for the studied traits (except chamfer and umbilical hernia) have been efficient throughout the study period.

## Data Availability

The datasets analyzed during the current study are available from the corresponding author upon request.
